# Pycnodysostose: à propos d’un cas

**DOI:** 10.11604/pamj.2018.31.93.8388

**Published:** 2018-10-08

**Authors:** Abdelhakim Elyajouri, Mohammed Benyahia, Rachid Abilkassem, Aomar Agadr

**Affiliations:** 1 Service de Pédiatrie, Hôpital Militaire d’Instructions Mohamed V, CHU Ibn Sina, Rabat, Maroc

**Keywords:** génétique, pycnodysostose, pediatrie, Osteopathy, pycnodysostosis, pediatrics

## Abstract

La pycnodysostose est une maladie osseuse génétique très rare associant une ostéocondensation, un syndrome dysmorphique et un retard de croissance. Rappeler les anomalies phénotypiques, les signes radiologiques, la prise en charge thérapeutiques et évolutifs de la Pycnodysostose chez un enfant de 11 ans. Enfant de 11 ans, a été réferé par son dentiste pour évaluation de son état clinique. Il est de parents consanguin de premier degré, qui présentait depuis l'âge de 3 ans des fractures spontanées à répétition. L'examen trouvait un syndrome dysmorphique fait de bosse frontale, fontanelle antérieure persistante, micrognathie, malformations des doigts, malposition dentaire, ongles incurvées, thorax asymétrique, attitude scoliotique du rachis dorsal, avec retard statural important (-4DS). Les radiographies du squelette montraient une densification des os de la base du crâne, une persistance de la fontanelle antérieure, une malposition dentaire, une densification diaphysaire et métaphysaire des os longs prédominants au niveau des membres inférieurs avec présence des cals vicieux et des phalanges grêles des mains. L'ostéodensitométrie était normale. Devant les signes cliniques et les manifestations radiologiques, le diagnostic retenu est une pycnodysostose. Un conseil génétique a été proposé pour la famille ainsi qu'une prise en charge dentaire et orthopédiques. La pycnodysostose est une entité rare de diagnostic parfois difficile et tardif, elle pose un problème du diagnostic avec l'ostéoporose. Le traitement est essentiellement préventif des fractures et des caries dentaires.

## Abstract (en)

Pycnodysostosis is a very rare genetic disease of the bone characterized by osteocondensation associated with dysmorphic syndrome and growth retardation. This study aims to highlight the phenotypic abnormalities, the radiological signs, the therapeutic and evolutionary features of pycnodysostosis in a 11-year old child. The child was referred by his dentist for clinical evaluation. He was born to first-degree consanguineous parents and had recurrent spontaneous fractures since the age of 3 years. Clinical examination showed dysmorphic syndrome characterized by frontal hump, persistent anterior fontanelle, micrognathia, finger deformities, dental malposition, curved nails, asymmetric chest, lumbar spine scoliosis with severe growth retardation (-4DS). Skeletal X-rays showed bony densification of the skull base, persistent anterior fontanelle, dental malposition, diaphysometaphyseal densification of the long bones mainly at the level of the lower limbs with malunions and tapered phalanges of the hands. Bone densitometry was normal. The diagnosis of pycnodysostosis was retained based on the clinical and radiological signs. Genetic counselling was proposed to the family as well as dental and orthopaedic treatment. Pycnodysostosis is a rare disorder; diagnosis is sometimes difficult and delayed posing diagnostic problem due to its resemblance with osteoporosis. Treatment is essentially based on fractures and dental caries prevention.

## Introduction

La pycnodysostose, ou syndrome de toulouse-lautrec, est une ostéopathie condensante héréditaire transmise sous une forme autosomique récessive [1]. L'anomalie responsable de la maladie est située sur le gène de la cathepsine K (1q21) [2]. L'objectif de notre travail est de rappeler les anomalies phénotypiques, les signes radiologiques, la prise en charge thérapeutiques et évolutifs de la Pycnodysostose chez un enfant de 11 ans.

## Patient et observation

**Figure 1:**
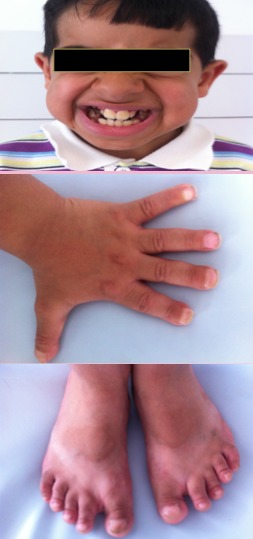
Le syndrome dysmorphique de notre patient

**Figure 2:**
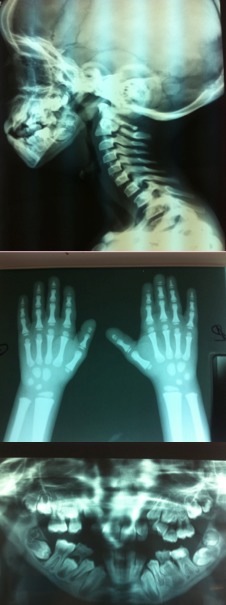
Les anomalies osseuses de notre cas

Enfant de 11 ans, referé par son dentiste pour évaluation de son état clinique. Il est de parents consanguin de premier degré, qui présentait depuis l'âge de 3 ans des fractures spontanées à répétition, prédominante au niveau des deux tibias. L'examen trouvait un syndrome dysmorphique fait de bosse frontale, fontanelle antérieure persistante, micrognathie, malformations des doigts, malposition dentaire avec de multiples caries, ongles incurvées, thorax asymétrique, attitude scoliotique du rachis dorsal, avec retard statural important (-4DS) (Figure 1). Les radiographies du squelette montraient une densification des os de la base du crâne, une persistance de la fontanelle antérieure, une malposition dentaire, une densification diaphysaire et métaphysaire des os longs prédominants au niveau des membres inférieurs avec présence des cals vicieux, et des phalanges grêles des mains (Figure 2). L'ostéodensitométrie était normale. Devant les signes cliniques et les manifestations radiologiques, le diagnostic retenu est une pycnodysostose. Un conseil génétique a été propose pour la famille ainsi qu'une ne prise en charge dentaire et orthopédiques.

## Discussion

La pycnodysostose est une ostéopathie condensante héréditaire très rare, touchant les deux sexes. Sa prévalence est de 1/ 100 000 [3]. De transmission autosomique récessive, la consan- guinité parentale est décrite chez 30% des cas [4]. Les arguments permettant de porter le diagnostic sont: cliniques, avec retard statural prédominant aux membres qui sont courts et massifs, fontanelle ouverte ou se ferment tardivement, dysmorphie faciale caractéristiques (macrocéphalie, saillie des bosses frontales et occipitales, micrognathie, hypoplasie faciale avec nez proéminent, koïlonychie, défaut d'implantation dentaire avec double rangée) [5]. À la radiographie, l'ostéosclérose diffuse est généralisée. Les fractures sur traumatisme de faible énergie sont fréquentes et peuvent être multiples. Le crâne est marqué, comme le cas de notre patient, par la présence d'une hypoplasie du maxillaire inférieur et des os de la face avec défaut de pneumatisation des sinus et de la mastoïde. La dysplasie ostéolytiques des phalanges distales et l'hypoplasie de l'extrémité acromiale de la clavicule sont typiques [6]. L'ostéopétrose demeure le principal diagnostic différentiel de la pycnodysostose. Elle s'en distingue par: la taille normale, l'absence de modelage métaphysaire donnant l'aspect « en massue », striation de la métaphyse des os longs et des corps vertébraux, condensation des plateaux vertébraux « vertèbre en sandwich », la fréquence des complications infectieuses, nécrotiques, hématologiques et des compressions des nerfs crâniens [7].

## Conclusion

La pycnodysostose est une entité rare de diagnostic parfois difficile et tardif, elle pose un problème du diagnostic avec l'ostéoporose. Le traitement est essentiellement préventif des fractures et des caries dentaires. Le traitement curatif est celui des complications (fractures et extraction dentaire).

## Conflits d’intérêts

Les auteurs ne déclarent aucun conflit d'intérêts.

## References

[1] Hodder A, Huntley C, Aronson KJ, Ramachandran M (2015). Pycnodysostosis and the making of an artist. Gene.

[2] Puri R, Saxena A, Mittal A, Arshad Z, Dwivedi Y, Chand T (2013). Pycnodysostosis: an anaesthetic approach to this rare genetic disorder. Anesthesiology.

[3] Mujawar Q, Naganoor R, Patil H, Thobbi AN, Ukkali S, Malagi N (2009). Pycnodysostosis with unusual findings: a case report. Case J.

[4] Alves Pereira D, Berini Aytes L, Gay Escoda C (2008). Pycnodysostosis: a report of three clinical cases. Med Oral Patol Oral Cirugia Bucal.

[5] Sudarshan R, Vijayabala GS (2012). Pycnodysostosis'a review. SEAJCRR.

[6] Ramaiah K, George GB, Padiyath S, Sethuraman R, Cherian B (2011). Pyknodysostosis: report of a rare case with review of literature. Imaging Sci Dent.

[7] Abourazzak S, Chaouki S, Akodad Z, Boubou M, Atmani S, El Biaz M (2013). Sleep apnea and short stature revealing pycnodysostosis. Arch Pediatr.

